# Hemodynamical consequences and tolerance of sustained ventricular tachycardia

**DOI:** 10.1371/journal.pone.0285802

**Published:** 2023-05-17

**Authors:** Hubert Delasnerie, Caroline Biendel, Meyer Elbaz, Franck Mandel, Maxime Beneyto, Guillaume Domain, Quentin Voglimacci-Stephanopoli, Pierre Mondoly, Clement Delmas, Vanina Bongard, Anne Rollin, Philippe Maury

**Affiliations:** 1 Department of Cardiology, University Hospital Rangueil, Toulouse, France; 2 I2MC, INSERM UMR 1297, Toulouse, France; The Open University, UNITED KINGDOM

## Abstract

**Aims:**

Factors underlying clinical tolerance and hemodynamic consequences of monomorphic sustained ventricular tachycardia (VT) need to be clarified.

**Methods:**

Intra-arterial pressures (IAP) during VT were collected in patients admitted for VT ablation and correlated to clinical, ECG and baseline echocardiographical parameters.

**Results:**

114 VTs from 58 patients were included (median 67 years old, 81% ischemic heart disease, median left ventricular ejection fraction 30%). 61 VTs were untolerated needing immediate termination (54%). VT tolerance was tightly linked to the evolution of IAPs. Faster VT rates (p<0.0001), presence of resynchronization therapy (p = 0.008), previous anterior myocardial infarction (p = 0.009) and more marginally larger baseline QRS duration (p = 0.1) were independently associated with VT tolerance. Only an inferior myocardial infarction was more often present in patients with only tolerated VTs vs patients with only untolerated VTs in multivariate analysis (OR 3.7, 95% CI 1.4–1000, p = 0.03). In patients with both well-tolerated and untolerated VTs, a higher VT rate was the only variable independently associated with untolerated VT (p = 0.02). Two different patterns of hemodynamic profiles during VT could be observed: a regular 1:1 relationship between electrical (QRS) and mechanical (IAP) events or some dissociation between both. VT with the second pattern were more often untolerated compared to the first pattern (78% vs 29%, p<0.0001).

**Conclusion:**

This study helps to explain the large variability in clinical tolerance during VT, which is clearly related to IAP. VT tolerance may be linked to resynchronization therapy, VT rate, baseline QRS duration and location of myocardial infarction.

## Introduction

Clinical tolerance of sustained monomorphic ventricular tachycardia (VT) is variable and hemodynamical consequences of VT need to be clarified. Although VT rate is involved in hemodynamical tolerance, other mechanisms were described for explaining hemodynamical behavior during VT, such as atrio-ventricular dissociation [1, 2] and the uncoordinated ventricular contraction because of the ectopic pattern of ventricular activation [3–5]. Blurred or compensatory neuro-hormonal response to baroreceptor activation [2, 6] have been demonstrated. Finally, myocardial ischemia might explain some additional decrease in systolic function [7].

Currently, there are no clear individualized predictable factors correlating to hemodynamical consequences and clinical tolerance during monomorphic VT. Predicting VT tolerance in a given patient would have some clinical interest, for example for optimizing ICD programmation, favoring anti-tachycardia pacing maneuvers or immediate ICD shocks according to the expected VT tolerance, or for predicting if sustained episodes will be tolerated during VT ablation. This would also be of interest when selecting patients who will best benefit of an ICD, since this may be postponed when well-tolerated VTs are expected.

In this study, we analyzed invasive arterial pressures and their evolution during VT and correlated these with clinical features, ECG and echocardiographic parameters.

## Methods

We performed a retrospective study of 114 VTs recorded in 58 successive patients referred for VT ablation at the University Hospital of Toulouse in the second half of 2019. Patients were referred for VT ablation either for an elective procedure (n = 37) or for electrical storm (n = 21). Patients with incessant or refractory VTs were not included, because hemodynamical behavior in this critical setting is not representative of stable balanced conditions, and any analysis would be furthermore precluded by the lack of stable baseline control conditions because of ongoing/incessant VTs. Patients with left ventricular assist techniques were not included.

For each patient, cardiac history, underlying cardiomyopathy, cardio-vascular risks factors, coronary status, medical therapy, symptoms and ECG were retrospectively collected. The most recent data was used regarding echocardiography or coronary angiography (performed during the same hospitalization, except if recent data over the preceding weeks or months was available).

Patients were studied in a fasting state under light sedation using morphine and midazolam (4 and 2 mg respectively in each patient). No general anesthesia was used in any patient. Implantable cardioverter-defibrillators (ICD) were deactivated in implanted patients. Standard programmed ventricular stimulation–i.e. 3 basic rates, up to 3 extrastimuli up to 200 ms coupling interval–was performed during the ablation procedure, either for documenting VT morphology and/or performing activation mapping, or after completion of the ablation process for determining the success of the procedure. Only sustained monomorphic VTs were kept for analysis (i.e. VT lasting > 30 seconds or terminated before because of immediate intolerance). Spontaneous sustained VT or VTs induced incidentally during catheter manipulation were also included in analysis. One or more sustained VTs were included for each patient.

Polymorphic VTs or VF were not included (because of cardiac arrest during VF or because of the non-sustained or quickly degenerating nature of polymorphic VT), as well as VTs induced under isoproterenol (because of vasodilatator and inotropic properties and because of the non-clinical conditions). Furthermore, even if sustained, polymorphic VT would preclude analysis based on cycle length and QRS morphology.

Patients under vasopressive drugs were excluded. Anti-arrhythmic drugs were not interrupted before the procedure.

### ECG measurements

Standard 12 lead ECG was recorded on the electrophysiological system (LabSystem Pro, Boston Scientific ^TM^) at 1 mV/mm amplification, with 0.05–150 Hz filters settings and 1 KHz sampling rate.

Baseline cardiac rhythm, rate, QRS duration and morphology/axis were noted. Baseline QRS duration was measured either on native QRS (for those not paced at baseline before VT induction) or on paced beats (for those constantly paced before VT induction).

Cardiac rate, QRS duration and morphology/axis were also analysed during VT, together with presence of concordance (i.e., fully positive or negative QRS complexes in all the precordial leads) and atrio-ventricular dissociation (AV dissociation) as visible on surface ECG. Unstable VTs, carrying the risk of degenerating into less tolerated and more risky arrhythmias, were defined by spontaneous deterioration in VF or by spontaneous change in VT morphology and/or rate.

### Hemodynamical measurements

Invasive arterial pressure (IAP) was constantly monitored during the whole procedure. Pressure transducer was connected to the sheath introduced into the femoral artery. For retrograde aortic access (n = 44), IAP was measured on the 9 French long sheath used for mapping and ablation, while a 5 French short sheath was inserted in the femoral artery in patient with transseptal access (n = 9). Air bubbles were flushed and the zero level was defined as the pressure measured by the external transducer positioned at the expected level of the right atrium in the supine position. Arterial pressure curve was stored together with 12-lead ECG on the electrophysiological recording system. Measurements were performed retrospectively on screen.

Baseline IAP was defined by systolic arterial pressures measured during the last beats before the train of stimuli inducing VT or before spontaneous VT onset. IAP during VT was defined by systolic arterial pressures measured at 10, 30 seconds, 1, 3, 5 and 10 minutes after VT onset. Minimal IAP and delay until minimal IAP was noted, as well as late increase in IAP (or late IAP drop when present) and IAP before VT termination (last VT beats). Delay to IAP recovery after VT termination (until IAP reached the baseline value) was also noted. IAP values were averaged over three successive beats when variable.

Untolerated VT was defined as VT needing urgent termination by overdrive or external DC-shock because of clinical signs indicating very low cardiac output, which may carry immediate severe prognosis if not immediately interrupted (loss of consciousness or other severe warning signs such as confusion, seizures, or sustained IAP ≤ 40 mm Hg). Otherwise, VT was let ongoing, allowing activation mapping and was terminated either spontaneously or when full mapping was done or by ablation. In case of spontaneous or overdrive-induced change in VT morphology and/or VT rate, the initial VT was considered terminated and no further IAP measurements were done (no IAP recovery available in these cases) and the subsequent VT–occurring or induced during the course of the first one—was not included in analysis.

Three types of analysis were performed:

- Comparison between tolerated and untolerated VTs in the whole population.
- Comparison between patients with only tolerated VTs versus patients with only untolerated VTs.
- Paired comparisons between tolerated and untolerated VTs in patients presenting both VTs (data averaged when several VTs in the same group).

According to the French ethics and regulatory law, retrospective studies based on the exploitation of usual care data shouldn’t be submitted to an ethical committee but have to be covered by reference methodology of the French National Commission for Informatics and Liberties (CNIL). This study was approved by the Toulouse University Hospital, which confirmed that ethical requirements were totally respected (number RnIPH 2021–83).

### Statistics

Continuous variables were reported as median and interquartile range (IQR) and compared with unpaired t-tests. Categorical variables were compared using Chi-square tests. For paired comparisons, the paired-t test or Wilcoxon’s test were used for continuous variables, and the McNemar test for the categorical ones. Repeated measurements were compared with ANOVA. The Spearman test was used for establishing correlations between variables.

Logistic regressions were performed for determining the parameters associated with untolerated VT. All the parameters significantly related to VT tolerance in univariate analysis were considered as eligible explanatory variables in a full multivariate logistic model. A conditional logistic regression analysis was conducted on the 22 patients who experienced both well-tolerated and untolerated VTs to identify explanatory variables significantly and independently associated with VT tolerance.

A score for predicting VT tolerance was proposed, including parameters independently linked to VT tolerance. Categorical variables were dichotomized (yes/no) and numerical values were grouped in classes. Weighting of each parameter was based on the β value from the multivariate logistic regression, and null values were chosen for those indicated to be associated with best VT tolerance.

Analysis and calculations were performed using SAS V9.4 (SAS Institute, Cary NC). A p value < 0.05 was considered statistically significant.

## Results

### 1. Population characteristics

Fifty-eight successive patients with sustained monomorphic VTs were retrospectively included, allowing the analysis of 114 fully analyzable VTs. Clinical, ECG and echocardiographic characteristics of the patient’s population are depicted in Table 1.

**Table 1:** Characteristics of the study population (n = 58).

*Male gender*	*54/58 (93%)*
*Age (years)*	*67 (IQR 17)*
*Height (m)*	*1*.*74 (IQR 0*.*95)*
*Weight (kg)*	*84 (IQR 22)*
*BMI (kg/m2)*	*28 (IQR 6)*
*Body surface (m2)*	*2*.*03 (IQR 0*.*3)*
*NYHA class*	*2 (IQR 1)*
*Dyslipidemia*	*30/58 (52%)*
*Diabetes*	*12/58 (21%)*
*Hypertension*	*28/58 (48%)*
*Obesity (BMI > 30 kg/m2)*	*13/58 (22%)*
*Smokers*	*35/58 (60%)*
*History of supraventricular arrhythmias*	*20/58 (34%)*
*Severe chronic renal failure*	
*(creatinine clearance <30ml/min)*	*2/58 (3%)*
*Previous Stroke*	*5/58 (9%)*
*Ischemic heart disease*	
*(previous myocardial infarction)*	*47/58 (81%)*
*Previous coronary angioplasty*	*29/43 (67%)*
*Previous coronary by-pass*	*5/47 (11%)*
*Right coronary artery patent = 24*	
*stenosis = 10*	
*occlusion = 10*	
*Circumflex/marginal artery patent = 25*	
*stenosis = 15*	
*occlusion = 3*	
*Left descending artery/left coronary artery patent = 13*	
*stenosis = 26*	
*occlusion = 5*	
*Previous inferior MI*	*15/43 (35%)*
*Previous lateral MI*	*6/43 (14%)*
*Previous anterior MI*	*25/43 (58%)*
*One vessel disease*	*19/44 (43%)*
*Two vessels disease*	*14/44 (32%)*
*Three vessels disease*	*9/45 (20%)*
*Beta-blocker*	*52/58 (90%)*
*Amiodarone*	*46/58 (79%)*
*Beta-blocker + Amiodarone*	*43/58 (74%)*
*Sotalol*	*2/58 (4%)*
*Calcium-Channel blockers*	*1/58 (2%)*
*Lidocaine*	*5/58 (8%)*
*Vasodilatators*	*52/58 (90%)*
*Diuretics (furosemide)*	*28/58 (48%)*
*Furosemide daily dosing (mg)*	*80 (IQR 85)*
*mineralocorticoid receptor antagonists 19/58 (33%)*	
*ICD (transvenous)*	*42/58 (72%)*
*Cardiac Resynchronization Therapy*	*13/58 (22%)*
*Clinical presentation of the VT*:	
*Cardiac arrest*	*5/58 (8%)*
*Syncope*	*16/56 (29%)*
*Acute Heart failure*	*13/58 (22%)*
*Palpitations*	*14/54 (26%)*
*Chest pain*	*7/58 (12%)*
*Electrical storm*	*21/58 (36%)*
*Baseline ECG*:	
*Sinus rhythm*	*50/58 (86%)*
*Heart rate (bpm)*	*70 (IQR 28)*
*QRS duration (ms)*	*128 (IQR 48)*
*Left superior axis*	*24/58 (41%)*
*Right inferior axis*	*24/58 (41%)*
*Right superior axis*	*7/58 (12%)*
*Left inferior axis*	*3/58 (5%)*
*Left Bundle branch block*	*4/58 (7%)*
*Right Bundle branch block*	*9/58 (16%)*
*Paced (right ventricle)*	*6/58 (10%)*
*Paced (bi-ventricular)*	*13/58 (22%)*
*Undetermined intra-ventricular block 7/58 (12%)*	
*Narrow QRS*	*19/58 (33%)*
*Echocardiography*:	
*LVEF (%)*	*30 (IQR 20)*
*LVEF < 35%*	*37/58 (64%)*
*Longitudinal strain (-%)*	*-10*.*5 (IQR 4)*
*Cardiac index (ml/min/m^2^)*	*1919 (IQR 898)*
*Cardiac index <2*.*2 ml/mn/m^2^*	*16/23 (69%)*
*End-diastolic LV volume (ml/m^2^)*	*101 (IQR 84)*
*Dilated left ventricle*	*34/48 (71%)*
*Left ventricular hypertrophy*	*2/56 (4%)*
*LV diastolic dysfunction*	*20/47 (43%)*
*Dilated right ventricle*	*8/50 (16%)*
*RV systolic dysfunction*	*18/52 (35%)*

Eighty-one percent had coronary artery disease cardiomyopathy, followed by valvular disease (n = 7), dilated cardiomyopathy (n = 6), hypertrophic cardiomyopathy (HCM) and arrhythmogenic right ventricular cardiomyopathy (ARVC) (n = 2 each), myocarditis and congenital heart disease (n = 1 each). Seven patients (12%) had mixed forms of cardiomyopathy. No patient had idiopathic VT. Coronary status was adequately managed before ablation in all patients with coronary artery disease. The clinical VT was judged untolerated (syncope or cardiac arrest) in 21 patients (36%).

### 2. VT characteristics

One hundred and fourteen sustained monomorphic VTs (median 2 per patient, one to five) were analyzed. VTs’ characteristics are depicted in Table 2.

**Table 2:** VT characteristics (n = 114).

*QRS duration (ms)*	*176 (IQR 40)*	
*VT rate (bpm)*	*176 (IQR 53)*	
*VT rate 100–149 bpm*	*29/114 (25%)*	
*VT rate 150–199 bpm*	*58/114 (51%)*	
*VT rate > 200 bpm*	*27/114 (24%)*	
*Concordance*	*25/114 (22%)*	
*Negative concordance*	*11/25 (44%)*	
*Positive concordance*	*14/25 (56%)*	
*Left inferior axis*	*6/114 (5%)*	
*Left superior axis*	*45/114 (39%)*	
*Right inferior axis*	*49/114 (43%)*	
*Right superior axis*	*14/114 (12%)*	
*Left bundle branch block pattern*	*51/114 (45%)*	
*Right bundle branch block pattern*	*63/114 (55%)*	
*AV dissociation*	*40/114 (35%)*	
*epicardial origin*	*22/114 (19%)*	
*Hemodynamical characteristics during VT*		
*Parameter*	*median (IQR)*	*Number of VT*
*Baseline IAP*	*115 (28)*	*114*
*10 sec IAP (mm Hg)*	*55 (38)*	*114*
*30 sec IAP (mm Hg)*	*71 (42)*	*84*
*1 min IAP (mm Hg)*	*82 (39)*	*58*
*3 min IAP (mm Hg)*	*82 (36)*	*30*
*5 min IAP (mm Hg)*	*88 (34)*	*20*
*10 min IAP (mm Hg)*	*88 (44)*	*13*
*Minimal IAP (mm Hg)*	*46 (26)*	*114*
*Minimal IAP (%)*	*44 (24)*	*114*
*IAP decrease (mm Hg)*	*61 (35)*	*114*
*IAP decrease (%)*	*56 (24)*	*114*
*Delay to minimal IAP (sec)*	*5 (5) (12 mm Hg/sec)*	*114*
*Delayed IAP increase (mm Hg)*	*13 (21)*	*114*
*IAP before VT termination*	*67 (49)*	*114*
*Delay to IAP recovery (sec)*	*2*.*7 (5)*	*82*

Median duration of VT was 60 sec (IQR 153). Twenty-three VTs were considered unstable (29%). VT morphology displayed a predominance of superior left and inferior right axis.

Concordant VTs were faster (192 IQR 30 vs 174 IQR 50 bpm, p = 0.05) and occurred more often in diabetic patients (11/26 vs 14/88, p = 0.004) but had similar QRS duration (176 IQR 64 vs 176 IQR 26 ms, p = ns).

### 3. Hemodynamical characteristics

**Fig 1:**
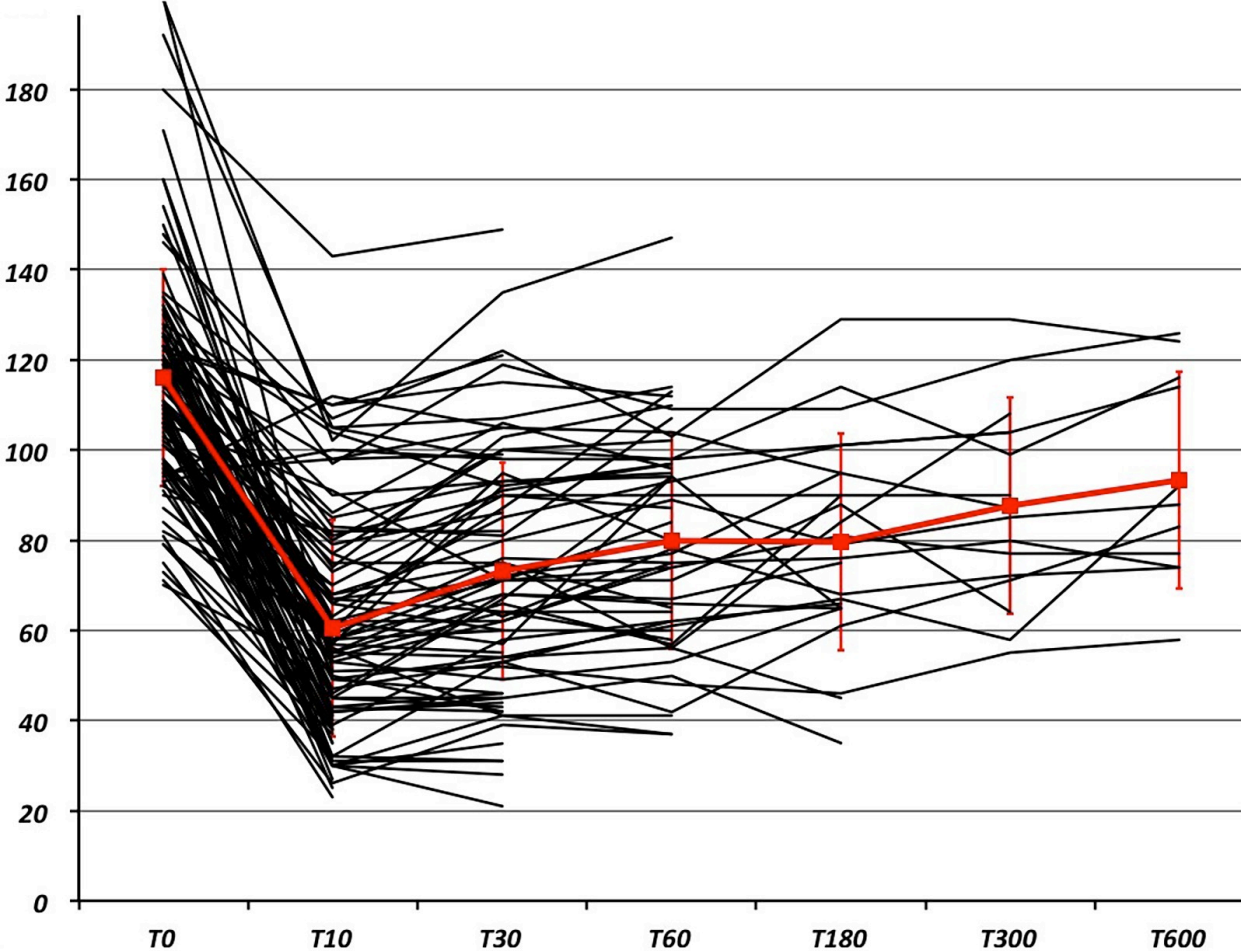
IAP profile for each VT at each time of evaluation (red: mean values). Horizontal axis = time in seconds after VT onset. Vertical axis = IAP (mm Hg).

**Fig 2:**
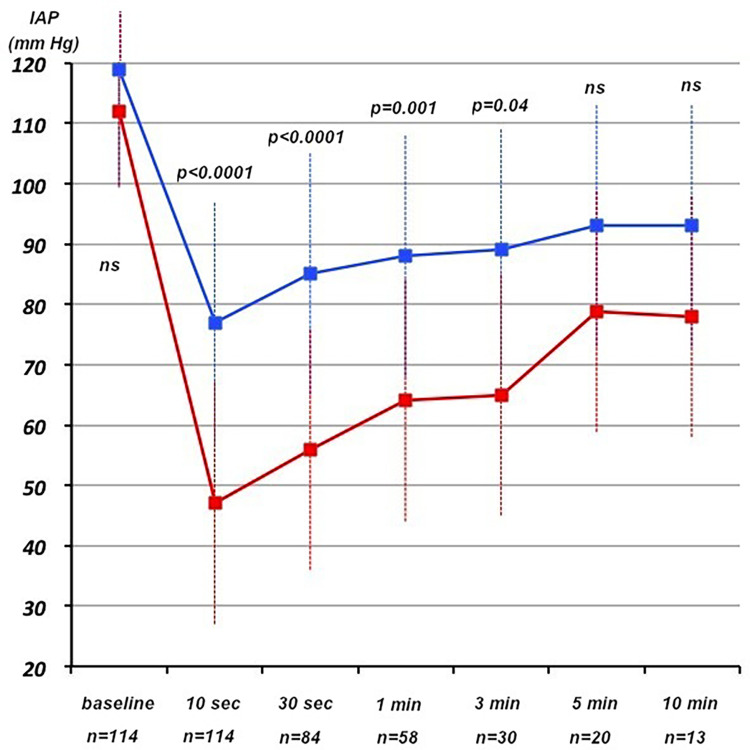
Mean IAP profile correlated to the tolerance of VT.

Evolution of IAP during VT can be found in Table 2 and shown in Figs 1 and 2.

Minimal IAP < 50 mm Hg was noted in 54%, minimal IAP < 90 mm Hg in 94%, and a IAP decrease > 50 mm Hg in 68%. A delayed increase in IAP was usually noted once a minimal value was reached. Late IAP drop was conversely observed in 9 VTs at various timings (mean decrease 12 mm Hg, IQR 14). Except for less amiodarone (55 vs 85%, p = 0.02), there was no difference between VTs with late drop and the other ones.

VT was considered untolerated in 54% (n = 61/114). Termination of untolerated VTs happened 31sec after VT onset (IQR 30). In 54 of these 61 cases, VT termination was elicited shortly after VT onset (28 sec, IQR 19), while it was delayed in seven cases (540 sec, IQR 528).

### 4. Comparisons

#### A. Comparisons between tolerated and untolerated VTs in the whole population (n = 114 VTs)

Untolerated VTs were significantly associated with a lower minimal absolute or relative IAP during VT, as well as higher absolute and relative drop in IAP, more drop > 50 mm Hg and more minimal IAP < 50 or 90 mmHg during VT, but there was no difference in baseline IAP. Untolerated VTs were of shorter durations (because being quickly terminated for obvious reasons p = 0.003) and displayed slower (p = 0.03) but more continuous and regular IAP drop which ultimately reached lower values compared to well-tolerated VTs. IAP was significantly lower in untolerated VTs during the first three minutes, but did not differ significantly thereafter for VTs of sufficient duration (although number of such VT was low) (Fig 2), except for the last VT beats before termination. Delayed increase of IAP was higher for well-tolerated VTs. There was no correlation with the presence of late IAP drop or unstable episodes. Delay to IAP recovery was longer in case of untolerated VTs.

Relevant differences between the 61 untolerated VTs and the 53 well-tolerated VTs can be found in S1 Table. In univariate analysis, a faster VT rate, the presence of concordance on ECG, a decreased longitudinal strain, presence of ICD or resynchronization therapy and location of previous MI were significantly linked to untolerated VT. Borderline associations were also found with previous acute coronary syndrome or angioplasty (p = 0.1) or lesions of left descending artery (p = 0.07), larger baseline QRS duration (p = 0.06), the absence of diabetes (p = 0.08) and more previous stroke (p = 0.07) in untolerated VTs. There was no significant association with other parameters.

On multivariate analysis including some of the most relevant parameters (excluding longitudinal strain due to the low number of values), a faster VT rate, presence of resynchronization therapy, an anterior MI and more marginally a larger baseline QRS duration remained significantly and independently associated with untolerated VTs (Table 3).

**Table 3:** Relevant parameters related to VT tolerance after multivariate analysis (n = 114 VT).

	*OR*	*95% CI*	*p value*
*VT rate*	*1*.*06*	*1*.*03–1*.*09*	*<0*.*0001*
*Resynchronization therapy*	*16*	*2*.*05–125*	*0*.*008*
*Anterior vs infero-lateral MI*	*7*.*3*	*1*.*6–32*.*2*	*0*.*009*
*Baseline QRS duration*	*1*.*02*	*0*.*99–1*.*04*	*0*.*11*
*VT Concordance*	*3*.*14*	*0*.*55–17*.*8*	*0*.*19*
*Diabetes*	*0*.*63*	*0*.*16–3*.*42*	*0*.*59*

#### B. Comparisons between patients with only tolerated VTs and patients with only untolerated VTs

When the 20 patients with only untolerated VTs were compared to the 16 patients with only well-tolerated VTs, no significant difference could be found, except less diabetes, less inferior MI and less electrical storm in patients with only untolerated VTs (and marginally a lower LVEF, lower cardiac output, lower longitudinal strain, more anterior MI and more mineralocorticoid receptor antagonist) (Table 4).

**Table 4:** Significant or borderline difference between patients with only tolerated VTs vs only untolerated VTs.

*Parameters*	*Only*	*Only*	*Total patients*	*p value*
	*tolerated VT*	*untolerated VT*		
	*(n = 16)*	*(n = 20)*	*(n = 36)*	
*Inferior MI*	*7/14 (50%)*	*1/15 (7%)*	*8/29 (27%)*	*0*.*009*
*Diabetes*	*6 (37%)*	*1 (5%)*	*7 (19%)*	*0*.*01*
*Electrical storm*	*8 (50%)*	*4 (20%)*	*12 (33%)*	*0*.*05*
*LVEF (%)*	*40 (IQR 20)*	*26 (IQR 22)*	*34 (IQR 20)*	*0*.*07*
*Anterior MI*	*6/14 (43%)*	*11/15 (73%)*	*17/29 (59%)*	*0*.*09*
*Mineralocorticoid receptor antagonist (MRA)*				
	*3 (18%)*	*9 (45%)*	*12 (33%)*	*0*.*09*
*Longitudinal strain (%)*				
	*-12 (IQR 7)*	*-9 (IQR 7)*	*-11 (IQR 5)*	*0*.*1*
*Low Cardiac Index (<2*,*2 l/min/m2)*				
	*5/11 (45%)*	*8/10 (80%)*	*13/21 (62%)*	*0*.*1*
*Cardiac Index (l/min/m2)*				
	*2*.*5 (IQR 0*.*9)*	*1*.*6 (IQR 1*.*3)*	*2 (IQR 1)*	*0*.*16*

Only an inferior MI was significantly linked to the group of patients with only tolerated VTs in multivariate analysis (OR 3.7, 95% CI 1.4–1000, p = 0.03).

#### C. Comparisons between untolerated and well-tolerated VTs in the same patients

In twenty-two patients, there were both untolerated and well tolerated VTs, allowing paired comparisons on a same patient-basis, with a total of 65 VT episodes (35 untolerated VTs versus 30 well tolerated VTs). Minimal IAP were lower, with larger IAP drop and a longer delay until minimal IAP in untolerated VTs, and VT rate, VT QRS duration and ECG concordance were significantly linked to VT tolerance. There was no correlation with QRS morphology/axis, AV dissociation or presence of late IAP drop (Table 5).

**Table 5:** Paired comparisons between well-tolerated and untolerated VTs in the same patients.

	*well-tolerated*	*untolerated*	*p value =*
	*VT (n = 35)*	*VT (n = 30)*	
*IAP decrease (mm Hg)*	*51 (IQR 30)*	*72 (IQR 25)*	*<0*.*0001*
*IAP decrease (%)*	*43 (IQR 18)*	*64(IQR 14)*	*<0*.*0001*
*Minimal IAP (mmHg)*	*64 (IQR 27)*	*41 (IQR 22)*	*<0*.*0001*
*Minimal IAP (%)*	*58 (IQR 25)*	*35 (IQR 14)*	*<0*.*0001*
*Delay to minimal IAP (sec)*	*4*.*2 (IQR 2*.*2)*	*7*.*8 (IQR 5*.*3)*	*0*.*017*
*Unadjusted (univariate)*			
*VT rate (bpm)*	*152 (IQR 42)*	*191 (IQR 28)*	*0*.*016*
*VT QRS duration (ms)*	*176 (IQR 40)*	*181 (IQR 36)*	*0*.*06*
*VT concordance*	*18%*	*45%*	*0*.*079*
*Adjusted (multivariate)*			
*VT rate (bpm)*			*0*.*021*
*VT QRS duration (ms)*			*0*.*083*
*VT concordance*			*0*.*2*

A conditional logistic regression analysis was conducted on 17 these 22 patients who experienced both well-tolerated and non tolerated VT to identify explanatory variables significantly and independently associated with tolerance. Among the selected variables in univariate analysis (VT rate, VT QRS duration, concordance), VT rate was the only variable significantly and independently associated with tolerance (OR 0.96, 95% CI 0.93–0.99, p = 0.021), although VT QRS duration was close to the significance threshold (Table 5).

### 5. Score for predicting VT tolerance

Since VT rate, location of MI, baseline QRS duration and presence of resynchronization therapy were independently linked to VT tolerance, these parameters were included in the proposed score for predicting VT tolerance (see methods). The values scale from 0 to 11 (Table 6). A score > 7 was only present in case of untolerated VTs, while a score < 2 was only seen in case of well-tolerated VTs.

**Table 6:** Proposed score for predicting VT tolerance.

VT rate < 150 bpm	0
VT rate 150 to 169 bpm	1.5
VT rate 170 to 199 bpm	3
VT rate > 200 bpm	5
No resynchronization therapy	0
Resynchronization therapy	2.5
Inferior or lateral MI	0
Anterior MI	2
Baseline QRS duration < 130 ms	0
Baseline QRS duration 130 to 149 ms	0.5
Baseline QRS duration 149 to 169 ms	1
Baseline QRS duration > 170 ms	1.5

### 6. Types of hemodynamical profiles during VTs

**Fig 3:**
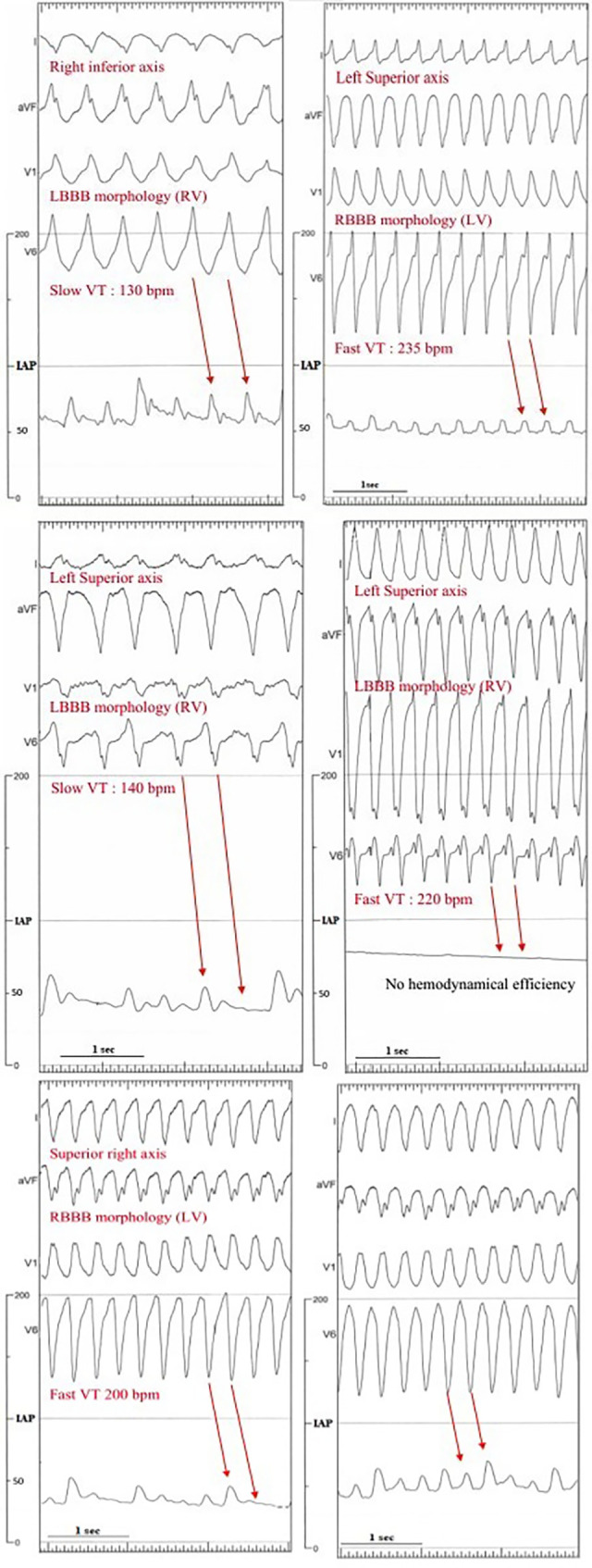
Upper: slow and fast well-tolerated VT with full 1:1 association between QRS and hemodynamical response. Middle: slow and fast untolerated VT with full or partial dissociation between QRS and hemodynamical response. Lower: example of transient dissociation between QRS and hemodynamical response followed by 1:1 association after one minute.

We could distinguish two different patterns of hemodynamic profiles during VTs: a preserved regular 1:1 relationship between electrical (QRS) and mechanical (IAP) events and some dissociation between them (Fig 3). When the second pattern was identified, VT was more often untolerated compared to the first one (78% vs 29%, p<0.0001). In a few patients, the second pattern was transiently observed, but after a time of adaptation, the first pattern then occurred (Fig 3).

## Discussion

This study using invasive evaluation gives some elements of understanding clinical tolerance and hemodynamical behavior during monomorphic sustained VT.

Although hemodynamical patterns during VT have been already described [3, 8], there are no clear predictable clinical factors of VT tolerance to date. This lack of evidence can be explained by the paucity of hemodynamical evaluation during VT. The few previous studies dedicated to this topic [6, 9–11] included a limited number of patients and concluded to an exaggerated decreased cardiac output during VT, especially in patients with impaired LV function, and sometimes to impaired baroreflex sensitivity to the initial drop of blood pressure. This work is the first one to gather abundant data in the context of VT ablation procedures, where IAPs are commonly and continuously monitored.

### General findings

In this study, VT tolerance was tightly linked to the IAP profiles. We found that faster VT rates, resynchronization therapy, anterior MIs and more marginally larger baseline QRS durations were independently associated with untolerated VTs in the whole population. We also found that myocardial infaction location was independently associated with VT tolerance, with inferior/lateral MI more frequently present in patients presenting with only tolerated VTs. Finally, faster VT rates during VT were independently associated with untolerated VTs when compared to well-tolerated VTs from the same patients.

Moreover, we described two phases of time-dependent hemodynamical profiles during sustained VT, with an initial phase with rapid IAP drop followed by a delayed slow IAP increase. Of note, a few patients presented late IAP drop again. These profiles were somewhat different between well-tolerated and untolerated VTs, with larger IAP drop, lower IAP and lower delayed IAP increase in untolerated VTs. Interestingly, initial IAP drop was faster in well tolerated VTs.

Finally, we also could describe two hemodynamical patterns correlated to VT tolerance, depending on the 1:1 relationship between QRS and IAP waves.

### Parameters linked to VT tolerance

Three main parameters are expected to condition hemodynamical consequences of sustained VT [3, 5, 10–14]: the baseline mechanical function of the heart, the cardiac rate during VT and the QRS characteristics during VT.

### 1. Baseline cardiac mechanical function

We found borderline lower LVEF and lower cardiac output in patients with only untolerated VTs, but these results were no more significant in multivariate analysis. Although we did not include healthy hearts in this study, a relevant proportion of patients had preserved LVEF, but interestingly this parameter did not seem to be relevant for hemodynamical tolerance of VT. The importance of LVEF in VT tolerance is debated, with different conclusions achieved in different populations [3, 7, 11, 12]. Increased spillover [15] and/or reduced uptake [16] of noradrenaline—as seen in case of ventricular dysfunction—may counterbalance the negative effects of low LVEF on hemodynamic during VT.

Other parameters related to left or right ventricular dimensions and mechanical performances were also not found to be linked to VT tolerance (except LGS but probably hampered by the low number of data).

We hypothetize that LVEF and other mechanical/anatomical parameters were unable to significantly correlate with VT tolerance possibly because hemodynamic is so altered during VT that differences in mechanical function or ventricular dimensions may have minor roles, and that even patients with preserved LVEF and anatomy may have undistinguishable altered IAP profiles. VT tolerance and hemodynamic are however still linked to some extent to the severity of underlying heart disease, as highlighted in this study by the higher proportion of resynchronization in the group of untolerated VTs, reflecting the selection of more diseased myocardium, regardless of LVEF, further altering tolerance during VT.

### 2. VT rate

VT rate was clearly higher in untolerated VTs, in the whole population, and also when tolerated VTs were compared to untolerated VTs in the same patients. This has been already mentioned [9, 12, 13, 17]. Reduced ventricular filling time, possible additional rate-induced altered diastolic function and reduced ejection according to Starling’s law easily explain dramatically low cardiac output during fast VT [3, 7]. However, VT rate cannot be considered as the unique parameter for tolerance [7, 8], an even slow VT are sometimes untolerated, especially in patients with very altered cardiac functions.

### 3. Anterior versus inferior MI location

Presence of an anterior versus inferior/lateral MI was also independently related to VT tolerance. This was probably due to the lower LVEF in these patients (which was not included in multivariate analysis since non significant in unadjusted comparisons), and additionally to larger baseline QRS duration (but which was independent in multivariate analysis).

### 4. ECG characteristics

Neither QRS duration, morphology or axis or AV dissociation were associated with VT tolerance in this study. While this did not seem to have been studied during VT, different findings have been made using different sites of ventricular pacing [3–5]. Enlarged QRS during VT are expected to desynchronize ventricular contraction [3, 4, 18, 19] and further alter cardiac output.

Interestingly, ECG concordance was associated with VT tolerance in the whole population, and when comparing untolerated vs tolerated VTs in the same patients, although no more after multivariate analysis. Normal activation through Purkinje network quickly depolarizes the myocardium, starting from septal endocardium to basal epicardium, for an optimized left ventricular ejection. Positive concordance corresponds to a posterior VT exit, so with an inverse depolarization vector. Negative concordance, corresponding to apical VT exit may be more physiological, but probably still with some major ventricular dyssynchrony, which needs to be confirmed. No separate analysis of positive or negative concordance was made due to the low number of cases. In this study, concordant VTs were faster, explaining the unadjusted association with VT tolerance.

Epicardial or endocardial locations of VTs were not associated with VT tolerance, although epicardial VT was expected to be less tolerated, because of a reverse pattern of depolarization and larger QRS, leading to more mechanical dyssynchronization. However, VT tolerance was not clearly associated to QRS duration during VT, and epicardial vs endocardial spreads of activation are difficult to demonstrate.

AV dissociation—as visually detectable on ECG—was not found predictive of VT tolerance, and there was no evident mechanical sign evocative of AV dissociation that can be suspected on IAP curves. If easy to detect during slowest VT rates, and suspected to have some hemodynamical role—for example in favoring mitral valve opening [1, 12]—it remains uncertain when undetectable AV dissociation would alter IAP in some ways, for example when slow bumps were seen on IAP curves. Further studies including atrial recordings during VT are needed for investigating this point.

### 5. Potential role of diabetes

Surprisingly, diabetes seemed to act as a protective factor, linked to better VT tolerance, although this was no more significant in multivariate analysis. Diabetes-induced impairment of the autonomic nervous system [20] with increase in sympathetic tone or up-regulation of beta-receptors because of sympathetic cardiac denervation may also explain this better tolerance, but these hypothesis remain to be demonstrated.

### Time-dependent hemodynamical profiles

Finally, we described the time-dependent hemodynamical profiles of sustained VT, with an initial rapid IAP drop over the first seconds, followed by a delayed slow IAP increase after around 30 seconds. This delayed progressive gain in IAP, probably caused by reflex vasoconstriction after activation of arterial baroreflex, may explain the recovery of consciousness after initial syncope as observed in clinical practice. A few patients also presented with a second late IAP drop sometimes needing VT termination. Previous studies also described a very similar two-steps process [9]. Interestingly, the slower the initial IAP drop, the lesser the VT was tolerated. Quick decrease in IAP may activate the baroreflex more vigorously [6].

### Two kinds of hemodynamical patterns

We also could describe two hemodynamical patterns correlated to VT tolerance, depending on the 1:1 relationship between QRS and IAP waves.

One of the hemodynamical patterns suggests some degree of full systolic inefficiency for some cardiac beats, which were more frequently observed in untolerated VTs, and may be considered as transient phases of electro-mechanical dissociations and possible harbinger of imminent lethal event. When the pattern changes during VT, it may signify that sympathetic tone is sufficient for allowing sudden better LV ejection and IAP rise [9]. Deeper analysis is needed for highlighting and understanding these patterns.

### Clinical applications

Predicting VT tolerance in a given patient would have some clinical interest. For example, optimizing ICD programming may be attempted by tailoring device programmation according to the factors of VT tolerance, favoring long and repeated sequences of painless anti-tachycardia pacing maneuvers in case of factors indicating good VT tolerance, while immediate ICD shocks may be delivered in the opposite cases. Besides, parameters linked to well-tolerated VT may be tested in the future for selecting patients who will best benefit of an ICD in primary or even secondary prevention.

### Limitations

Hemodynamical behaviour and clinical tolerance in the supine position cannot be extrapolated to stand-up position. Thus, our hemodynamical evaluation probably overestimates IAP values during VT in the real life, with a possible overdiagnosis of « well tolerated » VTs.

No general anesthesia was used in any patient. General anesthesia is leading to vasoplegy and hypotension and may render untolerated a VT which would have been better tolerated once awake. Since we only use light sedation, our findings are closer to more clinical “real life “situations. Despite morphine (and midazolam to a lesser extent) may have vasodilatator properties also, this effect is probably of minor importance, and this can not have biased our results since all patients received the same medications.

Myocardial ischemia was not actively screened as a potential factor of hemodynamical tolerance. However coronary artery disease was adequately managed in all patients before the ablation procedure, even if discrete residual ischemia could not be ruled out, which may have some undetermined consequences such as late IAP drops in some patients.

Scar burden or viability was not assessed in this study. If MRI may reveal different scar patterns or viability according to the VT tolerance deserves further study.

Baroreflex was not evaluated in this study. Sensitivity of baroreflex has been demonstrated for explaining clinical tolerance [6].

QRS amplitude alternans or R-R alternans were not analyzed, but, although interesting, no major form of QRS or RR alternans was present in our population.

Conditional logistic regression analysis was conducted on 22 patients only, thus one should remain prudent in interpretation of some borderline negative values (i.e. QRS duration during VT).

The proposed score for predicting VT tolerance should now be tested in independent populations.

## Conclusion

This study helps to explain the large variability in clinical tolerance during VT, which is clearly related to intra-arterial pressures. VT tolerance was independently linked to VT rate, presence of resynchronization therapy, location of previous myocardial infarction, presence of concordant VT and QRS duration during VT.

## Supporting information

S1 Table Relevant differences between the 61 untolerated VTs and the 53 well-tolerated VTs (no other parameter was statistically linked to VT tolerance). (DOCX)

S1 Data (ODS)
