# Supplementary material for: Hemodynamical consequences and tolerance of sustained ventricular tachycardia
Source: PLoS One. 2023 May 17;18(5):e0285802. doi: 10.1371/journal.pone.0285802 (PMC10191287; doi:10.1371/journal.pone.0285802)
Supplement: S1 Table — (DOCX) [file pone.0285802.s001.docx]

Supplementary table 1: Significant or borderline differences according to VT tolerance in the 114 VTs

| **Parameters:** | | |  | **Tolerated VT (n = 53)** | | **Untolerated VT (n = 61)** | | **Total**  **(n=114)** | | **p value** |
| --- | --- | --- | --- | --- | --- | --- | --- | --- | --- | --- |
| Diabetes | | |  | | 16 (30%) | | 10 (16%) | | 26 (23%) | 0.08 |
| History of stroke | | |  | | 2 (4%) | | 8 (13%) | | 10 (9%) | 0.07 |
| ICD | | |  | | 35 (66%) | | 50 (82%) | | 85 (74%) | **0.05** |
| Resynchronization Therapy | | |  | | 9 (17%) | | 20 (33%) | | 29 (25%) | **0.05** |
| Previous acute coronary syndrome * | | |  | | 29/43 (67%) | | 21/42 (50%) | | 50/85 (59%) | 0.1 |
| Previous coronary angioplasty * | | |  | | 29/43 (67%) | | 21/42 (50%) | | 50/85 (59%) | 0.1 |
| Left descending artery (patent/ stenosis/occlusion) | | |  | | 16/21/3 | | 10/23/10 | | 26/44/13 | 0.07 |
| Previous inferior myocardial infarction (MI) | | |  | | 18/39 (46%) | | 10/40 (25%) | | 28/79 (35%) | **0.05** |
| Previous inferior or lateral MI | | |  | | 22/39 (56%) | | 12/40 (30%) | | 34/79 (43%) | **0.02** |
| Previous anterior MI | | |  | | 19/39 (49%) | | 28/40 (70%) | | 47/79 (59%) | **0.05** |
| Infero-lateral vs anterior MI | | |  | | 20 vs 17 | | 10 vs 28 | | 30 vs 45 | **0.01** |
| Baseline QRS duration (ms) | | |  | | 128 (IQR 45) | | 144 (IQR 42) | | 144 (IQR 40) | 0.06 |
| Longitudinal strain (-%) |  | | | | -12 (IQR 3) | | -8 (IQR 7) | | -10 (IQR 5) | **0.04** |
| VT rate (bpm) | | |  | | 150 (IQR 36) | | 192 (IQR 41) | | 176 (IQR 53) | **<0.0001** |
| VT rate < 150 bpm | | |  | | 27 (51%) | | 6 (10%) | | 33 (29%) | **<0.0001** |
| VT rate > 200 bpm | | |  | | 5 (9%) | | 22 (36%) | | 27 (24%) | **0.0009** |
| VT concordance | | |  | | 7 (13%) | | 18 (30%) | | 25 (22%) | **0,04** |
| IAP decrease (mm Hg) | |  | | | 52 (IQR 33) | | 71 (IQR 28) | | 61 (IQR 35) | **0.0002** |
| IAP decrease (%) | | |  | | 43 (IQR 19) | | 65 (IQR 16) | | 56 (IQR 24) | **<0.0001** |
| > 50 mmHg IAP decrease | | |  | | 28 (53%) | | 50 (82%) | | 78 (68%) | **0.0008** |
| Delay to minimal IAP (sec) | | |  | | 3.8 (IQR 3.7) | | 6.9 (IQR 5.2) | | 5.2 (IQR 5) | **0.03** |
| Minimal IAP (mm Hg) | | |  | | 62 (IQR 30) | | 38 (IQR 16) | | 46 (IQR 26) | **<0.0001** |
| Minimal IAP (%) | | |  | | 57 (IQR 20) | | 35 (QR 17) | | 44 (IQR 24) | **<0.0001** |
| Minimal IAP < 50 mm Hg | | |  | | 14 (26%) | | 47 (77%) | | 61 (54%) | **<0.0001** |
| Minimal IAP < 90 mm Hg | | |  | | 45 (85%) | | 61 (100%) | | 106 (93%) | **0.001** |
| Late IAP increase (mm Hg) | | |  | | 23 (IQR 23) | | 8 (IQR 12) | | 13 (IQR 21) | **<0.0001** |
| IAP before VT termination (mmHg) | | |  | | 91 (IQR 38) | | 46 (IQR 24) | | 67 (IQR 49) | **<0.0001** |
| Delay to IAP recovery (sec) | | |  | | 1.7 (IQR 1.6) | | 5.4 (IQR 5.8) | | 2.7 (IQR 4.7) | **0.0002** |

* ischemic patients. IAP= intra-arterial pressures
